# Integration of tomato reproductive developmental landmarks and expression profiles, and the effect of *SUN *on fruit shape

**DOI:** 10.1186/1471-2229-9-49

**Published:** 2009-05-07

**Authors:** Han Xiao, Cheryll Radovich, Nicholas Welty, Jason Hsu, Dongmei Li, Tea Meulia, Esther van der Knaap

**Affiliations:** 1 Horticulture and Crop Science, The Ohio State University/OARDC, Wooster, OH 44691, USA; 2 Molecular and Cellular Imaging Center, The Ohio State University/OARDC, Wooster, OH 44691, USA; 3 Department of Statistics, The Ohio State University, Columbus, OH 43210, USA

## Abstract

**Background:**

Universally accepted landmark stages are necessary to highlight key events in plant reproductive development and to facilitate comparisons among species. Domestication and selection of tomato resulted in many varieties that differ in fruit shape and size. This diversity is useful to unravel underlying molecular and developmental mechanisms that control organ morphology and patterning. The tomato fruit shape gene *SUN *controls fruit elongation. The most dramatic effect of *SUN *on fruit shape occurs after pollination and fertilization although a detailed investigation into the timing of the fruit shape change as well as gene expression profiles during critical developmental stages has not been conducted.

**Results:**

We provide a description of floral and fruit development in a red-fruited closely related wild relative of tomato, *Solanum pimpinellifolium *accession LA1589. We use established and propose new floral and fruit landmarks to present a framework for tomato developmental studies. In addition, gene expression profiles of three key stages in floral and fruit development are presented, namely floral buds 10 days before anthesis (floral landmark 7), anthesis-stage flowers (floral landmark 10 and fruit landmark 1), and 5 days post anthesis fruit (fruit landmark 3). To demonstrate the utility of the landmarks, we characterize the tomato shape gene *SUN *in fruit development. *SUN *controls fruit shape predominantly after fertilization and its effect reaches a maximum at 8 days post-anthesis coinciding with fruit landmark 4 representing the globular embryo stage of seed development. The expression profiles of the NILs that differ at *sun *show that only 34 genes were differentially expressed and most of them at a less than 2-fold difference.

**Conclusion:**

The landmarks for flower and fruit development in tomato were outlined and integrated with the effect of *SUN *on fruit shape. Although we did not identify many genes differentially expressed in the NILs that differ at the *sun *locus, higher or lower transcript levels for many genes involved in phytohormone biosynthesis or signaling as well as organ identity and patterning of tomato fruit were found between developmental time points.

## Background

Plants display a diverse array of shapes, sizes and categories of fruit. Within the Solanaceae family fruit categories range from capsules, drupes, pyrenes, berries, to several other types of non-capsular dehiscent fruits [1]. Within one species such as tomato (*Solanum lycopersicum *L.), fruit morphology varies dramatically among cultivated accessions. The dramatic diversity in tomato fruit shape and size is due to domestication and continued selection for its fruit characters [2,3]. Fruit formation starts with the development of the floral meristem. Within the floral meristem, the expression of organ identity genes gives rise to the four whorls namely the sepals, petals, stamen and gynoecium. The coordinate spatial and temporal expression of several classes of homeotic genes specifies the identity of floral organs [4-7]. A class genes control sepal identity, A and B class genes specify the identity of petals, B and C genes define stamen identity, and C genes control carpel identity. The E class genes act redundantly in specifying the identity of floral whorls in combinations with the A, B and C genes [5-7].

After organ specification within the floral meristem, a complex growth patterning is observed in the fourth floral whorl comprising the gynoecium, which will become the fruit after fertilization of the ovules. Along the apical-basal axis, the developing tissue types of the gynoecium are the stigma, style, ovary and gynophore, whereas along the mediolateral axis of the ovary the valves or pericarp, septum or columella, placenta and ovules are formed. In fruit such as that of Arabidopsis, the gynoecium also includes two dehiscence-related tissues, replum and valve margin [8,9]. Combined with the organ and tissue identity genes, patterning is controlled by the expression of genes determining organ polarity [10]. A critical stage of fruit patterning occurs at fertilization which, when successful, results in seed formation. Fruit of most species will abort if there is none or limited fertilization and seed set. Phytohormones, particularly auxin and gibberellins (GA), play critical roles in fruit set and early growth triggered by pollination and fertilization. Auxin and GA can also induce parthenocarpic fruits by triggering pollination-independent fruit growth in several species including tomato [11-15].

Descriptions of flower and fruit developmental stages have been established for several species. The stages have been used to interpret gene function, and to determine the spatial and temporal expression of genes involved in organ identity and patterning. In addition, detailed descriptions of developmental stages are needed for comparative analyses to unravel genetic and molecular mechanisms that give rise to floral and fruit diversity. Ideally, these stages should describe key developmental events that are shared among flowering plant species, so that the landmarks could be compared and queried across databases using key morphological developmental features. Buzgo et al (2004) compared three distant angiosperm species and proposed ten floral landmark stages. These landmarks comprise "inflorescence formation and flower initiation", "sepal initiation", "petal initiation", "stamen initiation", "carpel initiation", "microsporangia formation", "ovule initiation", "male meiosis", "female meiosis", and "anthesis" [16], which have been adopted in studies of several other species [17,18]. However, key fruit landmark stages that are applicable across species have not been described to date. For example, whereas Arabidopsis fruit development is described in eight stages, tomato fruit development is described in four [19,20]. Phase I of tomato fruit development comprises ovary development ending with fertilization. Phase II describes early fruit growth following fertilization and spans cell division and early embryo development. Phase III spans cell expansion and embryo maturation. The final phase IV is the ripening phase [19]. Both cell division and elongation occur concomitantly in the different parts of the tomato fruit, thus these two phases are not well separated during growth of the organ [21,22]. More importantly, the stages described for Arabidopsis and tomato detail species-specific events that are not applicable across species. Therefore, the establishment of universally applied fruit developmental landmarks would allow comparative analysis of data obtained from different species.

Tomato, classified as a berry fruit, represents an excellent model for floral and fruit development and is used extensively in comparative studies within the Solanaceae family [2,3,19,23]. Whereas some information is known about the regulation of organ identity and specification [24-29], information about fruit patterning in Solanaceous species is rather limited. Varieties that differ in fruit morphology offer an important resource to further our understanding on its patterning. Fruit size and shape of tomato are controlled by major and minor QTL loci [2,3,30]. For some of these major QTL, the underlying genes are known. *SUN *and *OVATE *control fruit elongation and therefore affect patterning along the apical-basal axis [31,32]. *FW2.2 *and *FAS *control fruit mass via increases of the placenta area and locule number, respectively, and thus affect patterning along the medio-lateral axis [33,34]. *SUN *encodes a member of the IQD protein family [32]. The founding member of the IQD protein family AtIQD1 is localized in the nucleus and its overexpression leads to increases in glucosinolate production in Arabidopsis [35]. The high expression of *SUN *in tomato leads to elongated fruit, whichis hypothesized to control increases in secondary metabolites and/or hormone levels. In the near-isogenic lines (NILs) that differ at *SUN*, the most significant fruit shape changes occur after anthesis during fruit set [32]. However a detailed developmental time-course describing fruit shape changes that would aid in understanding the mechanism by which *SUN *acts has not been described. Moreover, an evaluation of flower and fruit expression profiles in the *S. pimpinellifolium *LA1589 background has not been performed to date.

In this study, we adopt the floral landmarks established previously [16], and also propose new landmarks of fruit development that are applicable across angiosperm plant species. These landmarks are superimposed onto the fruit shape changes controlled by *SUN *and combined with gene expression profiles of floral buds 10 days prior to anthesis, anthesis-stage flowers and fruit 5 days post pollination.

## Results

**Figure 1:**
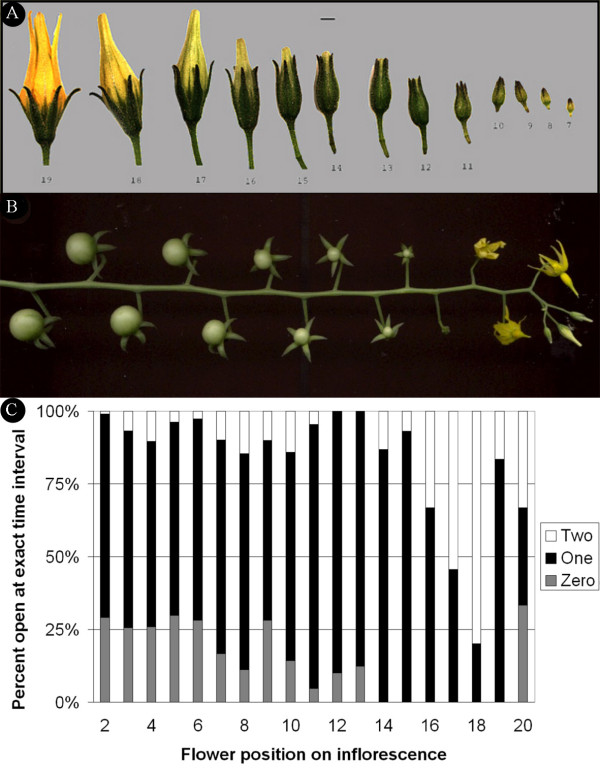
**Characterization of the *S. pimpinellifolium *accession LA1589 inflorescence**. (A) Series of consecutive floral buds 7 (right) to 19 (left) days since floral bud initiation. (B) Series of consecutive developing fruits on a given inflorescence. Note that two days after anthesis, the flower has senesced. (C) Timing of consecutive flower opening in LA1589 starting with the second oldest flower (2). The black bar indicates the percentage of flowers that opened at one-day time intervals at the position on the inflorescence listed on the X-axis. The white bar indicates the percentage of flowers that opened at two-day time intervals and the grey bar indicates the percentage of flowers that opened within the same day. Size bar represents 1 mm.

We used *S. pimpinellifolium *accession LA1589 for the tomato flower and fruit developmental studies due to its indeterminate growth habit and the abundant number of flowers and inflorescences throughout its life cycle. For example, LA1589 carries on average 20 flowers per inflorescence (Fig. 1A and 1B), whereas a typical cultivated variety carries only 3 to 7 flowers per inflorescence [36]. In addition, flower development is highly regular in the wild relative LA1589 compared to most cultivated types [36]. To time the developmental stages of consecutive buds and then fruits on an inflorescence, we recorded the time of anthesis for each flower in a total of 83 inflorescences investigated over four independent experiments. As shown in Figure 1C, the second flower opened 70% of the time one day after the first flower, 29% of the time on the same day as the first flower, and 1% of the time two days after the first flower and so on. In general, consecutive flower opening occurred at one-day intervals 75% of the time, until the 16th flower on a given inflorescence (Fig. 1C). Flower buds developed after the 16^th ^on a given inflorescence tended to open more irregularly and often at an interval of 2-days or more. By inference, this result implied that the first 16 floral meristems arose 75% of the time in one-day interval from one another. Therefore, we concluded that until the 16^th ^flower on a given inflorescence, the developing flower and fruit respectively, are staged at close to one-day intervals from one another.

### Initiation of floral organ primordia

**Figure 2:**
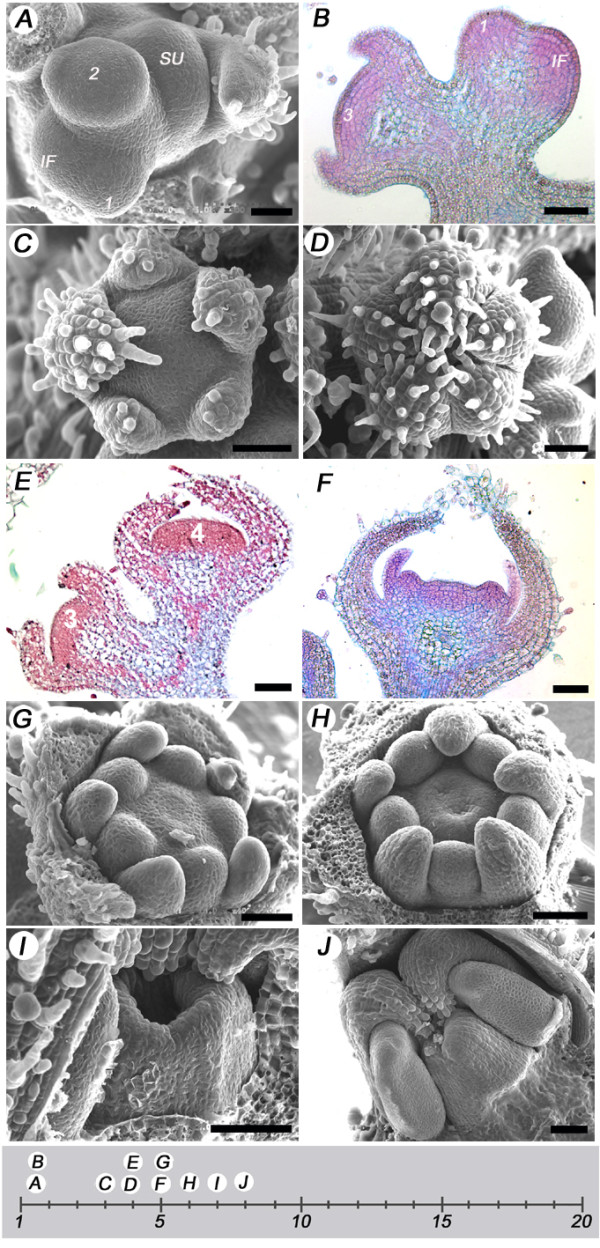
**Early flower developmental landmarks**. (A) Scanning electron microscopy image of a young inflorescence with the shoot meristem terminating into the inflorescence meristem, and the sympodial shoot meristem initiating the youngest leaf axil on the flank of the inflorescence, the youngest floral bud 1, and the second youngest bud 2 had also emerged from the inflorescence meristem. (B) Light microscopy image of a section from a young inflorescence showing the floral meristem, the youngest bud 1 and the third youngest bud 3. (C) Scanning electron microscopy images of a floral bud three days after flower initiation with sepal primordia, and (D) four days after floral initiation, with sepals enclosing over the floral meristem. (E) Light microscopy images of a section across two consecutive floral buds, three and four days after initiation, and (F) a floral bud six days after floral initiation, with petals and stamens emerging under the sepals. (G) Scanning electron microscopy images of floral buds at six days after floral initiation, with carpel primordia starting to emerge. The sepals were removed to visualize the developing petal, stamen and carpel. (H) Six days after floral initiation, with the central column rising and displaying the formation of the two locular cavities. (I) Seven days after floral initiation the carpel walls continue to elongate with the central column lagging behind. (J) Eight days after floral initiation, the ovary is closed and style has initiated. 1, youngest bud; 2, second youngest bud; 3, third youngest bud; 4, fourth youngest bud; IF, inflorescence meristem; SU, sympodial unit. Size bar in all images measure 50 μm.

The first landmark represented inflorescence formation and flower initiation (Table 1). The transition to flowering and inflorescence formation in LA1589 has been described previously [36]. Briefly, transition to flowering commenced with the termination of the vegetative meristem into an inflorescence meristem. Floral initiation occurred through the apparent bifurcation of the inflorescence meristem resulting in bud number 1 (Fig. 2A and 2B). The flatter inflorescence meristem continued its indeterminate growth pattern, while the more domed meristem developed into a flower (Fig. 2A and 2B). Following flower initiation, the emergence of the sepal primordia around the perimeter of the floral apex of bud number 2 marked the second landmark (Fig. 2A). The five tomato sepals initiated in a helical pattern of 144° (Fig. 2C). The sepals continued to grow and covered the floral meristem approximately 4 days after floral initiation (Fig. 2D and 2E). At the time of sepal enclosure, petal primordia started to arise, representing landmark 3. Following petal primordia emergence, stamen primordia emerged in alternate positions to the petals (Fig. 2F and 2G), at approximately 5 days after floral initiation, representing landmark 4. Sepals and petals continued to elongate while carpel primordia began to emerge in the floral center (Fig. 2G), marking landmark 5, which occurred approximately 6 days after floral bud initiation. The carpel walls or valves continued to enlarge, while the central part comprising the septum and the central column formed congenitally with the carpel walls, revealing the formation of the two locular cavities of wild type tomato ovary (Fig. 2H). The carpel walls elongated slightly faster than the central column revealing the locular cavity prior to ovary enclosure and initiation of the style, which occurred 8 days post bud initiation (Fig. 2I and 2J).

**Table 1:** Flower developmental landmarks.

**Flower Development Landmarks; Buzgo et al. (2004)**	**Days after flower initiation in tomato**	**Perianth organs**	**Reproductive organs**	
		
			**Ovary and ovule development**	**Stamen and pollen development**
(1) Inflorescence formation and flower initiation	1	Flattened inflorescence apex becomes dome-shaped.		

(2) Initiation of outermost perianth organs	2	Emergence of sepal primordia in a helical pattern.		

(3) Initiation of inner perianth organs.	4	Simultaneous emergence of petal primordia in alternating positions to the sepals. Sepals overlay the floral meristem		

(4) Stamen initiation	5	Sepals and petals elongate.		Simultaneous initiation of stamen primordia.

(5) Carpel initiation	6	Petals start curling over the stamens	Carpel primordia arise.	

	7		Central column that will form the locular cavities arise.	Stamen filament start developing and two anther lobes become visible.

(6) Microsporangia initiation	8		Central column continues to elongate. Carpels fuse at the apex of the ovary. Style initiation. Initiation of placental development.	Primary pariety cells develop into endothecium, middle layers and tapetum. Sporogenous layers visible.

(7) Ovule initiation	9		Ovule primordia begin to emergence from the placenta.	The two lobes of the anther and the locule are distinguishable, microsporocyte and tapetal cells are distinguishable. Binucleate tapetal cells.

(8) Male meiosis	10			Microsporogenesis. Microsporocytes or microspore mother cells undergo meiosis I and II and forming tetrads.

(9) Female meiosis	11		Megasporogenesis. Megaspore mother cell (meiocyte or megasporocyte) is visible. Meiosis I. The nucellus is small resulting in a tenui-nucellate ovule.	

	12	Petals grow to the top of sepals	The single integument begins to grow over the nucellus resulting in unitegmic ovules.	Callose wall surrounding the tetrads degrades releasing the microspores. Tapetum starts degenerating.

	13	Petals emerge from the sepals.	Micropyle development.	Free microspores are being incased in a thick polysaccharide wall; tapetum degenerated.

	14	Onset of sepal opening	Megagametogenesis and development of the embryo sac.	Microspores come vacuolated, and begins asymmetric mitosis

	15			Bi-cellular pollen grain.

	16		Ovule development nears completion.	The vegetative cell and generative cell are well distinguishable

(10) Anthesis	19	Petal opening		

The timing of the landmarks described by Buzgo et al (2004) in *S. pimpinellifolium *accession LA1589 floral development.

### Reproductive organ formation

**Figure 3:**
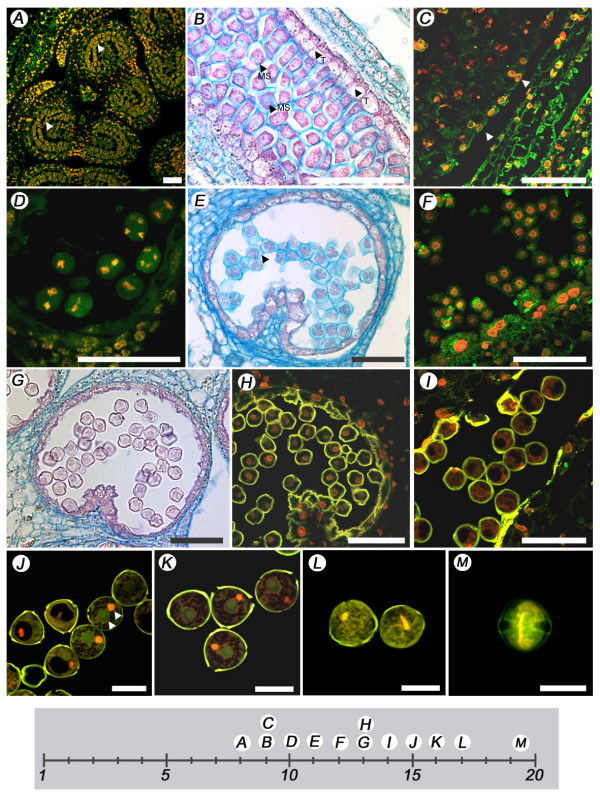
**Flower landmarks representing male reproductive development**. (A) Eight days after initiation, the primary sporogenous layers (arrowheads) have formed. (B) Nine days after floral initiation, the microsporocytes (MS) were visible in the sporogenous tissue as well as the tapetal cells (T). (C) Tapetal cells are binucleated (arrowhead). (D) Microsporocytes 10 days after floral initiation are undergoing meiotic divisions marking landmark 8. (E) Tetrads are enclosed by callose walls (arrowhead). (F) Release of microspores. (G – H) The tapetum is degenerating and the microspores are released 13 days after floral initiation. (I) The microspores become vacuolated 14 days after floral initiation. (J) Bi-cellular microspores. (K) Generative and vegetative cells are visible in microspores. (L) Seventeen days after floral initiation, the microspores show a crescent generative nucleus. (M) Pollen at anthesis. Scale bar, 50 μm (A-I), 20 μm (J-M).

Male reproductive development initiated with microsporangia development, which represented landmark 6, and occurred approximately eight days after floral bud initiation (Table 1 and Fig. 3A). The primary sporogenous layers were visible at this stage (Fig. 3A). Nine days after floral bud formation, the tapetal cells were binucleate, and the developing microsporocytes were also visible (Fig. 3B and 3C). At 10 days after floral bud initiation, microsporocytes or pollen mother cells were undergoing meiosis (Fig. 3D), marking landmark 8. A callose wall surrounded the four haploid nuclei of the tetrads (Fig. 3E). One day later, the callose walls began to degrade and the microspores were being released (Fig. 3F). At 13 days after floral bud initiation, the tapetum was degenerating; and the microspores were single and encapsulated in a thick wall (Fig. 3G and 3H). One day later, the microspores became vacuolated (Fig. 3I) and underwent one asymmetric mitosis. Fifteen days after floral bud initiation, the microspores were bi-cellular (Fig. 3J) and a day later, the generative and vegetative cells were clearly distinguishable within the developing pollen (Fig. 3K). At day 17 after floral bud initiation, the generative cell displayed the characteristic crescent shaped nucleus (Fig. 3L and 3M). The second mitosis of the generative cell did not occur until after pollination.

**Figure 4:**
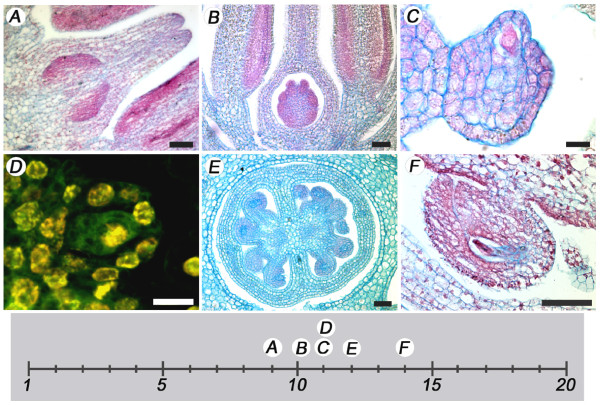
**Floral landmarks representing female reproductive development**. (A) Landmark 7 occurs nine days after floral initiation. (B) Ten days after floral initiation, the developing ovules become visible. (C) Eleven days after floral initiation, the megaspore mother cell forms, marking female meiosis and floral landmark 9. (D) Landmark 9 megaspore mother cell showing the nuclei (orange color) and the tubulin (green color). (E) Twelve days after floral initiation, the single integument has nearly covered the developing embryo sac. (F) The developing ovule with a clear micropyle is visible 14 days after floral initiation. Scale bar, 50 μm (A, B, F), 10 μm (C, D), 20 μm (E).

Female reproductive development initiated with the development of the ovules and represented landmark 7 (Fig. 4A). Approximately 9 days after floral bud initiation, the style and the ovary were nearly equal in length, and ovule primordia were emerging on the placental tissues (Fig. 4A). Ovules were clearly visible one day later (Fig. 4B). Two days after ovule primordia initiation and 11 days after floral bud initiation, a single integument started to envelope the single cell layered nucellus and the developing megasporocyte, resulting in a unitegmic tenui-nucleate ovule representing landmark 9 (Fig. 4C). Apparently the megasporocyte underwent the first meiotic division at this stage (Fig. 4D). A day later, the single integument at the base of the nucellus was clearly visible, while the megasporocyte is undergoing the second meiotic division, representing the first stage of megagametogenesis (Fig. 4E). Fourteen days after floral bud initiation, the integument enveloped the nucellus completely and the micropyle was well defined. The embryo sac development was taking place as evidenced by concentrated dark staining at the micropyle end. The presence of the megaspore at the chalaza end of the ovule indicated the development of the egg apparatus (Fig. 4F).

### Fertilization and fruit set

**Figure 5:**
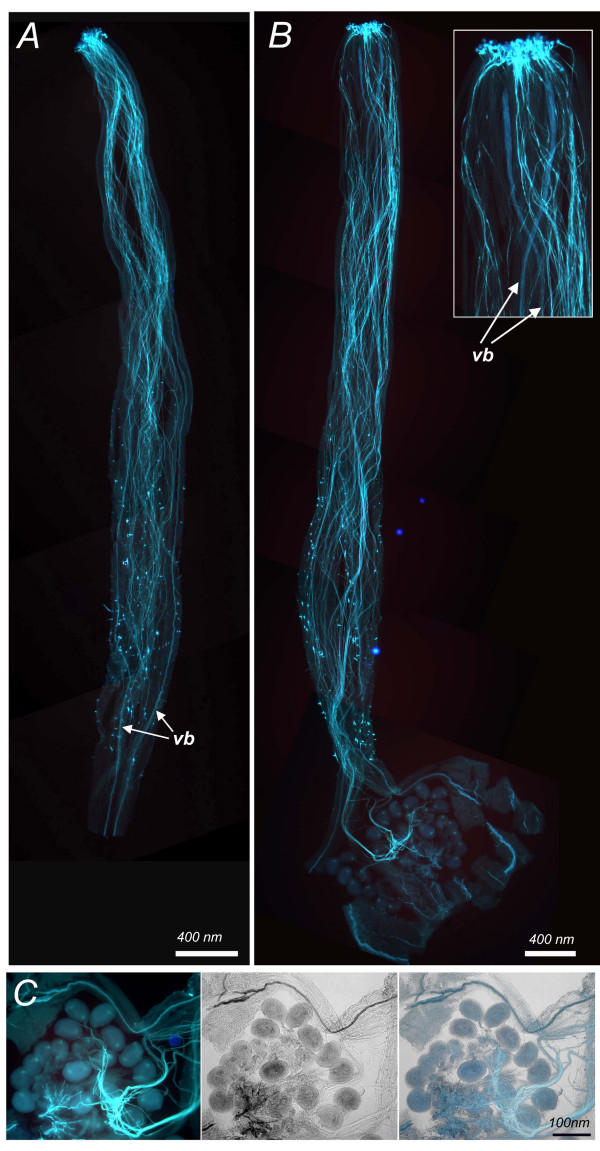
**Fertilization**. (A) Style at 6 hours after pollination. (B) Style at 10 hours after pollination. (C) Detail from an ovary at 10 hours after pollination. Several pollen tubes are penetrating the ovules. Scale bar, 400 μm (A and B), 100 μm (C). Styles were stained with aniline blue. VB, vascular bundles that fluoresce in a slightly different color blue compared to pollen tubes.

Anthesis or flower opening was the final floral landmark as well as the first fruit landmark (Table 1 and 2). At the time of anthesis, the anther lobes dehisced to release the pollen, which after landing on the receptive stigma, germinated. Pollen tubes had grown close to the base of the style 6 hours after pollination, and reached the ovules approximately 2 hours later (Fig. 5A and 5B). Ten to 12 hours after pollination, the pollen tubes had released their content resulting in fertilization of the ovules (Fig. 5C) and representing fruit development landmark 2 (Table 2). Senescence of floral organs, namely petal, stamens and style is associated with successful fertilization and was visible approximately two days after anthesis as shown in Fig. 1B.

**Table 2:** Fruit developmental landmarks.

**Fruit Development Landmarks**	**Days post anthesis**	**Descriptions of fruit development in tomato**	
		
		**Fruit growth (Gillaspy *et al *1993)**	**Embryo/seed development**
(1) Anthesis	0	Mature ovary, phase I.	Mature gametes. Pollen is shed, which will land on the stigma and germinate. Pollen tubes growth through the style.

(2) Fertilization	1–2	End of phase I, beginning of phase II.	Fusion of sperm and egg nuclei.

(3) 4–16 Cell Stage Embryo	3–6	Phase II and III, cell division and elongation stage.	First embryo divisions.

(4) Globular Stage Embryo	6–10	Phase III, cell expansion stage.	Globular embryo.

(5) Heart Stage Embryo	10–12	Phase III, cell expansion stage.	Heart Stage embryo lasts approximately one day and occurs 10–12 dpa.

(6) Torpedo Stage Embryo	13–16	Phase III, continued fruit enlargement.	Torpedo Stage embryo lasts approximately one day and occurs 13–16 dpa.

(7) Coiled Stage Embryo	20	Phase III, continued fruit enlargement.	Cotyledon expansion and curl as they elongate. Embryo appears physically mature, but the seed is not yet viable.

	20–28		Seed maturation period

(8) Seed germination	29–31	The fruit has reached the mature green stage. Fruit becomes sensitive to ethylene.	Seeds are becoming viable for germination.

(9) Fruit ripening	33–40	Ripening starts at the onset of the breaker stage. Changes in pigmentation are visible.	After ripening of seed.

(10) Ripe Fruit	40	Red ripe stage of tomato.	

Timing of the fruit landmarks in *S. pimpinellifolium *LA1589.

### Development of the pericarp after pollination

**Figure 6:**
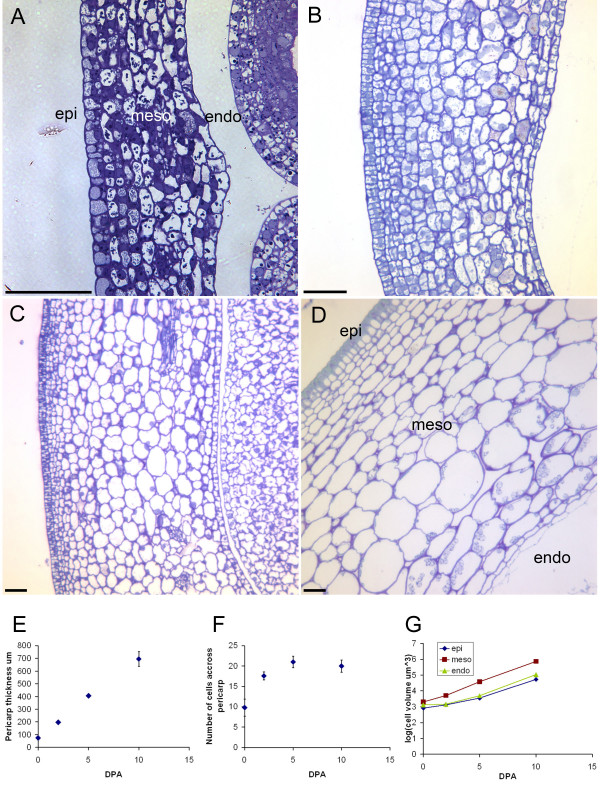
**Pericarp growth following anthesis**. (A) Pericarp at 0 dpa. (B) Pericarp at 2 dpa. (C) Pericarp at 5 dpa. (D) Pericarp at 10 dpa. (E) Thickness of the pericarp as a function of dpa. (F) Cell number across the pericarp as a function of dpa. (G) Cell size measured in the epicarp, mescocarp and endocarp was calculated from measured length (L) and width (W) using the following formula V = L*W*((L+W)/2). The log (volume) is plotted as a function of dpa. Epi, epicarp; meso, mesocarp; endo, endocarp. Size bar, 50 μm.

Following fertilization, tomato fruit growth consists of cell division and cell expansion [19]. We analyzed the growth of the pericarp following pollination to establish the timing of cell division and cell elongation in the developing LA1589 pericarp. Pericarp width doubled from anthesis to 2 days post anthesis (dpa), and then further doubled at 5 and 10 dpa, respectively (Fig. 6). Cell number across the pericarp increased from 10 at anthesis to 17 at 2 dpa, and reached the final number of 19–21 at 5 dpa (Fig. 6F), implying that cell division ended at or before that time. Mesocarp cell expansion started as early as 2 dpa (Fig. 6B). These results indicated that cell division and expansion occurred concurrently in the pericarp of the early developing fruit. Note the presence of the cuticle layer and starch granules in the epicarp and mesocarp respectively, of 10 dpa fruit (Fig. 6D).

### Seed development

As indicated above, cell division overlapped with cell elongation during the early stages of fruit development. Moreover, the cell division stage was short, ending before 5 dpa in LA1589, whereas the cell elongation stage spanned fruit development from 2 dpa until mature green stage. Thus, these two fruit developmental stages, which correspond to tomato development phases II and III, provided limited guides for referencing. To develop additional landmarks for the developmental stages of tomato fruit growth, we analyzed morphological changes in embryo development, which occur concomitantly with fruit growth in most angiosperm plant species.

**Figure 7:**
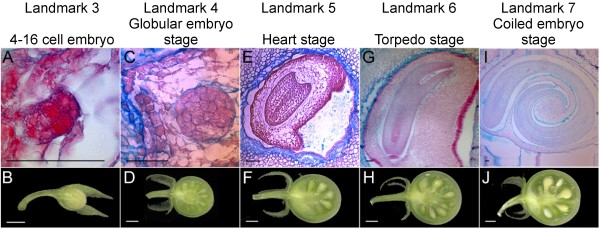
**Fruit landmarks described by stages of seed development**. (A-B) Landmark 3 corresponding to a 4 dpa fruit. (C-D) Landmark 4 corresponding to an 8 dpa fruit. (E-F) Landmark 5 corresponding to 10 dpa fruit. (G-H) Landmark 6 corresponding to 14 dpa fruit. (I-J) Landmark 7 corresponding to 20 dpa fruit. A, C, E, G, I are light microscopy sections stained with safranin O and astra blue. B, D, F, H, J show scanned fresh developing fruit images. Size bars are 50 μm for A, C, E, G, and I. Size bars are 2 mm for B, D, F, H, and J.

**Figure 8:**
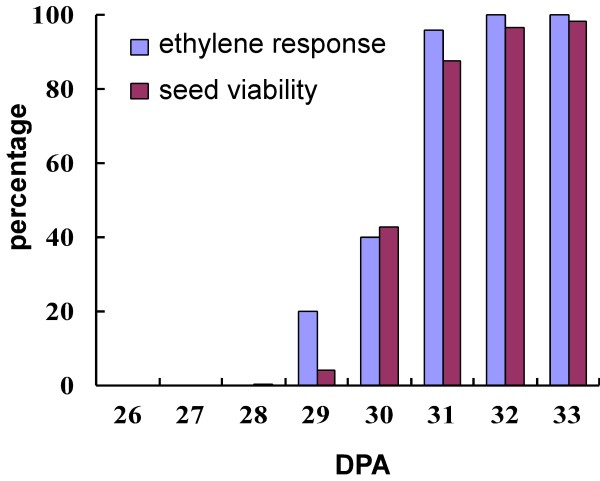
**Ethylene sensitivity of fruit and the corresponding seed viability**. The ethylene response and seed germination rate is plotted as a function of days post anthesis. Seed were extracted from half of the fruit prior to ethylene treatment of the remaining half.

We propose the third fruit developmental landmark as the stage of 4–16 celled embryo, which occurred approximately 4 dpa (Fig. 7A and 7B). The fourth landmark was represented by the globular embryo stage at 6 to 10 dpa (Fig. 7C and 7D). Heart shape embryo was the fifth landmark and occurred between 10 and 12 dpa (Fig. 7E and 7F) highlighting the beginning of cotyledon growth. The 13–16 dpa embryo was torpedo shape, marking the sixth landmark (Fig. 7G and 7H). After the sixth landmark, the cotyledons grew into a coil and reached the seventh landmark approximately at 20 dpa. At this stage, the embryo approached its final size, but the seed was not yet viable for germination (Fig. 7I and 7J). The eighth fruit developmental landmark was reached when the seeds harvested from the maturing fruit were viable for germination. Seed were collected from maturing fruit starting at 26 dpa until 33 dpa. Up until 29 dpa, there was little or no seed germination (Fig. 8). However, at 30 dpa, the germination rate increased dramatically thus reaching landmark eight. At 32 dpa, nearly 100% of the seeds germinated.

### Fruit ripening

Tomato fruit ripening stages consist of mature green, breaker and red ripe [19,23]. At the mature green stage, ethylene treatment will result in a rapid reddening of the fruit [23,37-39]. We measured ethylene sensitivity in half of the harvested fruits while determining the germination ability of the seed in the other half that were collected at selected times (see above). Ethylene sensitivity was achieved over a short period of up to two days, and coincided with the time when the seed became viable for germination (Fig. 8). Forty percent of fruit had responded to ethylene at 30 dpa when 43% of the seeds were viable for germination. Fruit younger than 29 dpa did not respond to ethylene treatment (Fig. 8). The ninth landmark is the onset of fruit ripening, coinciding with the breaker stage when color began to change at approximately 32 dpa. This stage is followed by the tenth and final landmark of ripe fruit.

### Gene expression profiles of floral and fruit development

To obtain a global overview of gene expression in flower and fruit, we compared the profiles between three critical developmental time points. The first stage was young flower buds at floral landmark 7, representing ovule initiation (10 days pre-anthesis). The second stage was the anthesis-stage, representing flower landmark 10 and fruit landmark 1. The third and last stage was 5 dpa fruits, representing the 4–16 cell embryo stage and fruit landmark 3. Differentially expressed genes were identified using the resampling-based multiple testing method [40]. Without the cutoff of fold-change applied, 2495 genes with adjusted *p *< 0.01 were differentially expressed in at least one of the three stages (see Additional file 1). Among them, 1232 genes showed higher expression at anthesis, whereas 527 and 736 genes showed higher expression in young flower buds and 5 dpa fruits, respectively (Table 3). Functional classification of the differentially expressed genes showed a distinct distribution of genes involved in various biological processes for the three stages investigated. For example, more genes involved in developmental processes were found in flower buds during ovule initiation and anthesis-stage flowers than in 5 dpa fruit. On the other hand, phytohormone-related genes were predominantly found in anthesis-stage flowers and 5 dpa fruits compared to flower buds (Table 3).

**Table 3:** Functional classification of differentially expressed genes during flower and early fruit development

	10 days preanthesis		Anthesis		5 DPA fruit	
Category	number	percentage	number	percentage	number	percentage

Cell cycle and Cell wall	14	2.66	20	1.62	14	1.90
Defense related	14	2.66	14	1.14	12	1.63
Developmental processes	32	6.07	74	6.01	16	2.17
Electron transport or energy pathway	11	2.09	27	2.19	13	1.77
Phytohormone related	10	1.90	48	3.90	21	2.85
Metabolism and other cellular processes	174	33.02	398	32.31	287	38.99
Regulation of transcription	17	3.23	43	3.49	21	2.85
Response to stimuli	25	4.74	63	5.11	26	3.53
Signal transduction	9	1.71	18	1.46	11	1.49
Structural	51	9.68	26	2.11	122	16.58
Transport	26	4.93	106	8.60	39	5.30
Unclassified	144	27.32	395	32.06	154	20.92
Total	527	100	1232	100	736	100

### Expression of organ identity and patterning genes

Of the genes representing the developmental processes, key floral and fruit patterning genes were examined for their expression profiles during reproductive development (see Additional file 2, Fig. 9). Genes orthologous or homologous to the Arabidopsis ABCE genes required for floral organ identity have been identified in tomato [41,42]. On our array, the tomato floral organ identity genes differentially expressed at the three stages include B class genes *TAP3 *(TC116723) [26], *TPI *(TC117703) and *SlMBP1/LePI-B *(TC119919) [42], C class gene *TAG1 *(TC124766) [43], and E class gene *TM29 *[44]. The tomato ortholog TC121763 of Arabidopsis *NAP *that is directly activated by B class gene *APETALA3 *and *PISTILLATA *in Arabidopsis [45] was also differentially expressed. All the above-mentioned genes showed higher expression in floral buds and/or anthesis-stage flowers (see Additional file 2), in agreement with their previously identified expression patterns. Another tomato B class gene *TM6 *(TC117238) was not differentially expressed, likely due to its more ubiquitous expression in floral organs [26]. While there is no clear ortholog of Arabidopsis A class genes in tomato [42], the closest related *AP1 *gene, *MADS-MC *(TC118643) [46], showed no expression changes in the three developmental stages. Many of the organ identity genes encode MADS box proteins of MIKC-type, and *in vitro *interaction analysis of twenty-two tomato MADS box proteins show modified as well as novel interaction patterns that had evolved for the family members in this species [47].

**Figure 9:**
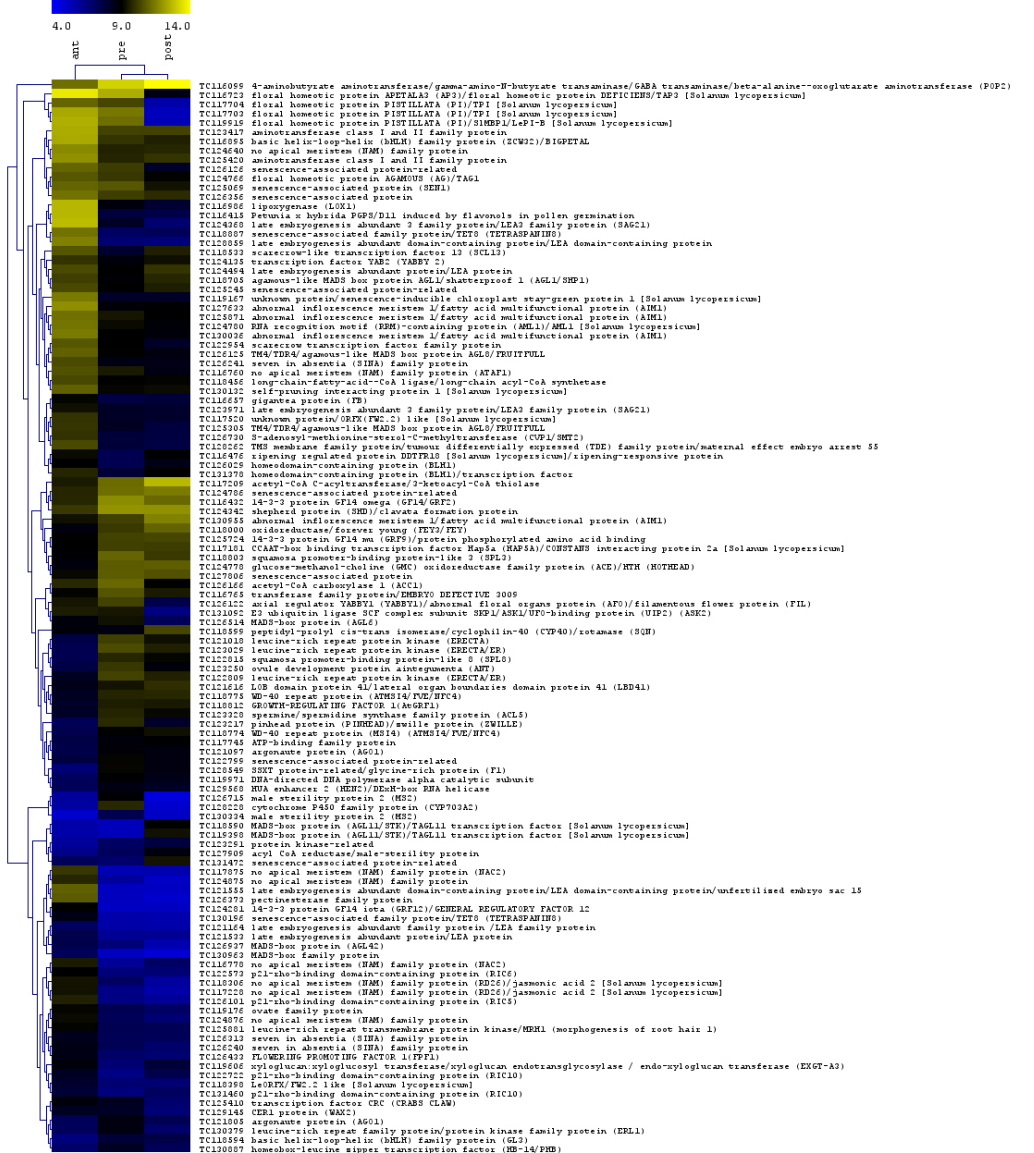
**Hierarchical clustering of expression for the differentially expressed genes involved in developmental processes**. Differentially expressed genes putatively involved in developmental process were selected by *multtest *package in R. Shown is the heat map representation for averaged expression intensities. All data are presented as log_2 _(RMA expression value).

In addition to these organ identity genes, other genes play key roles in patterning of the fruit. In Arabidopsis, these include the apical-basal patterning genes: *ETTIN *(*ETT*) [48], *LEUNIG *(*LUG*) [49], *TOUSLED *(*TSL*) [50], *STYLISH *(*STY1 *and *STY2*) [51], *SPATULA *(*SPT*) [52], *NO TRANSMITTING TRACT *(*NTT*) [53], and *HECATE *(*HEC1, HEC2 *and *HEC3*) [54], involved in basal valve growth, carpel and septum fusion, elongation of apical tissues, and style and transmitting tract formation, respectively. There are also genes patterning valve and valve margin of the fruit along the medio-lateral axis, including *SHATTERPROOF *(*SHP*) [55], *ALCATRAZ *(*ALC*) [56], *INDEHISCENCE *(*IND*) [57], *REPLUMLESS *(*RPL*) [58], and *FRUITFULL *(*FUL*) [59]. The Arabidopsis gene *SEEDSTICK *(*STK*) is required for ovule identity and patterning as well as seed disposal [60], and *ERECTA (ER) *regulates fruit shape by controlling cell expansion and cell division [61]. *JAGGED *(*JAG*) acts redundantly with the polarity genes *FILAMOUS FLOWER *(*FIL*) and *YABBY3 *(*YAB3*) to activate *FUL *and *SHP *[10]. Additional polarity genes required for proper patterning and establishment of organ boundaries are *CRABS CLAW *(*CRC*) [62], *KANADI *(*KAN1 *and *KAN2*) [63], *GYMNOS *(*GYM*) [64], *PHAVOLUTA (PHV) *and *PHABULOSA *(*PHB*) [65]. Tomato genes homologous to Arabidopsis patterning genes *FIL *(TC126122), *FUL *(TC125305 and TC126125), *CRC *(TC125410), *ER *(TC121018, TC122809 and TC123029), *PHB *(TC130887), and *SPT *(TC126307) were more abundantly expressed in tomato flower buds compared to the other tissues. The tomato *SHP *homolog TC118705 showed higher expression in anthesis-stage flowers and fruits at 5 dpa than in floral buds. The *STK *homolog in tomato *TAGL11 *(TC119398), which is expressed in the inner integument of the ovules and the endothelium in developing seeds [41], was expressed higher in fruits at 5 dpa compared to other time points (see Additional file 2), suggesting that it may also play a role in tomato fruit development. Tomato genes with high similarity to Arabidopsis fruit patterning genes *ETT, GYM, KAN2, LUG, PHV, RPL, HEC1, STY1 *and *TSL *were not differentially expressed between the three stages, whereas no tomato homologs for *JAG*, *NTT*, *ALC*, *IND*, *YAB3*, *STY2 *were included on our array. Further, the hierarchical clustering of all the 122 differentially expressed developmental processes genes revealed that flower bud and 5 dpa fruit shared expression profiles of the same developmental genes, whereas anthesis-stage flower showed a distinctive profile (Fig. 9, see Additional file 2), which is in agreement with results from other gene profiling studies in Arabidopsis [66-68].

### Expression of phytohormone-related genes

**Figure 10:**
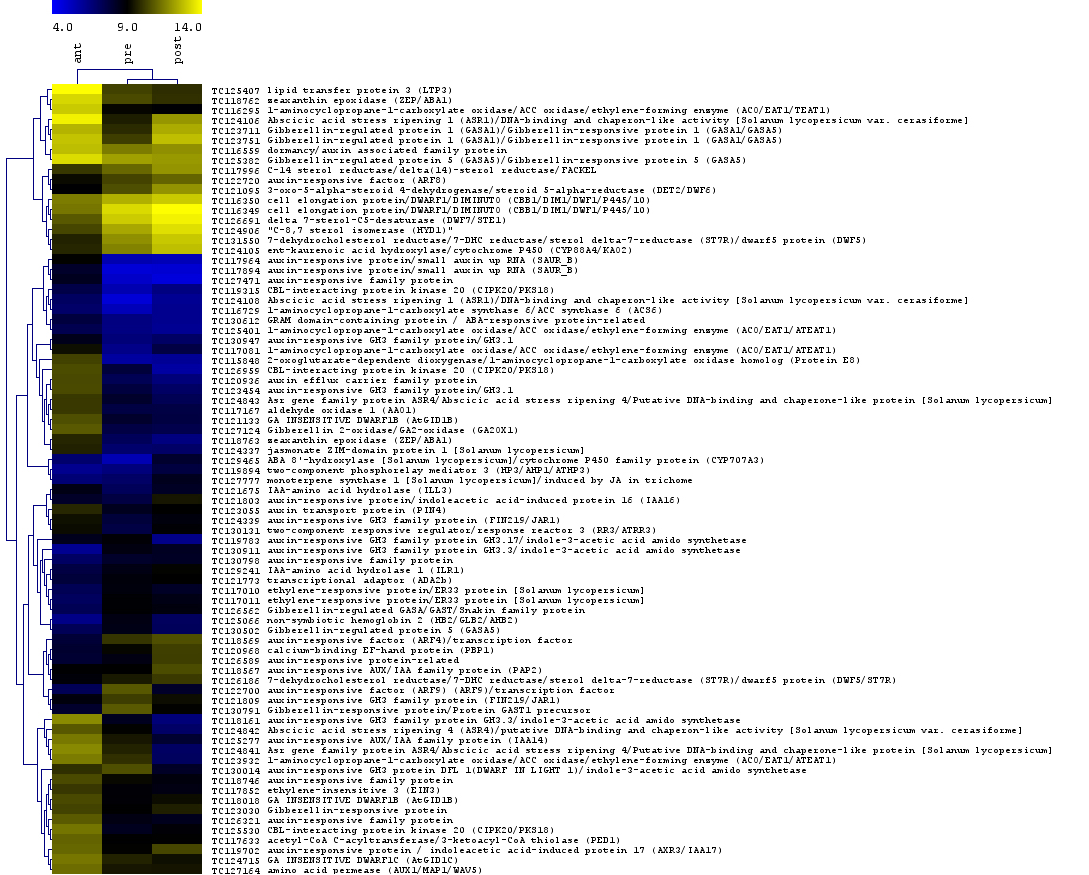
**Hierarchical clustering of expression for the differentially expressed genes involved in plant hormone biosynthesis and signaling**. Differentially expressed genes related to hormone were selected by *multtest *package in R. Shown are heat map representations for averaged expression intensities. All data are presented as log_2 _(RMA expression value).

Phytohormones play essential roles in many aspects of plant development. Among the three developmental time points, 79 phytohormone-related genes were differentially expressed (see Additional file 3). Of these genes, 30 were involved in auxin conjugation, transport or signaling. Most of the auxin-related genes (22 of 30) were either up- or down-regulated in 5 dpa fruit (Fig. 10, see Additional file 3). Moreover, most of the genes with similarity to *GH3 *involved in IAA conjugation were repressed after pollination, whereas three auxin response factor genes TC118569 (*ARF4*), TC122720 (*ARF8*), and TC122700 (*ARF9*), were expressed at the lowest level in anthesis stage flowers. Further, transcripts of three auxin transporter genes, TC127164, TC123055 and TC120936, homologous to *AUX1*, *PIN4 *and an auxin efflux carrier family protein, respectively, were less abundant in 5 dpa fruit (Fig. 10, see Additional file 3). Several genes involved in biosynthesis of tryptophan (TC119571, TC121695, TC125473, TC127841, TC129375, and TC130235), a precursor of IAA, were not developmentally regulated in this study, neither was the ortholog of Arabidopsis auxin receptor *TRANSPORT INHIBITOR RESPONSE1 *(*TIR*1, TC121284) [69]. The ortholog of *ALDEHYDE OXIDASE 1 *(*AAO1*, TC117167) involved in auxin biosynthesis [70], was expressed at higher level in anthesis flower. This may imply that many components in auxin pathway are channeled to the increasing demand for auxin-dependent programs to fulfill rapid fruit growth after pollination.

Some GA-related genes were also differentially expressed in the three developmental stages. Transcript levels of the tomato ortholog TC124105 of *AtKAO2 *that catalyzes the conversion of *ent*-kaurenoic acid to GA_12 _in gibberellin biosynthesis pathway [71], was more abundant in 5 dpa fruit compared to other stages. In contrast, the expression of *SlGA2ox2 *(TC127124), involved in catabolism of GA [72], was lower in the developing fruits than in flower buds at 10 days preanthesis and anthesis-stage flowers. Interestingly, transcripts of three tomato homologs TC118018, TC121133 and TC124715 of Arabidopsis GA receptors *GA INSENTIVE DWARF1B and C *(*GID1B and GID1C*) [73], were less abundant in 5 dpa fruit. This suggests that although GA levels may increase in 5 dpa fruit as a result of increased biosynthesis and reduced catabolism, the sensitivity to the hormone may decrease as a result of reduced expression of the receptor. GA biosynthesis genes of the GA 20-oxidase and GA 3-oxidase families were either not differentially expressed (*SlGA20ox-3, SlGA3ox-2*) or not included on the array (*SlGA20ox-1, -2 *and *SlGA3ox-1, -3*). Most of the seven GA responsive genes were not differentially expressed following pollination with the exception of tomato gene TC126562 encoding GASA/GAST/Snakin family protein that was upregulated after anthesis (Fig. 10, see Additional file 3).

Transcripts of all the eight brassinosteroid-related genes were more abundant in 5 dpa fruit, whereas the majority of jasmonate- and ethylene-related genes were less abundant in 5 dpa fruit (see Additional file 3). Expression of genes involved in ABA biosynthesis and response like were also lower in 5 dpa fruits. The putative ortholog of Arabidopsis gene *CYP707A3 *(TC129465), encoding the major ABA 8'-hydroxylase involved in ABA catabolism [74], is expressed at higher level in 5 dpa fruit compared to the other stages, suggesting that the ABA levels are reduced during the early fruit growth.

### Fruit shape changes in LA1589 NILs differing at *sun*

**Figure 11:**
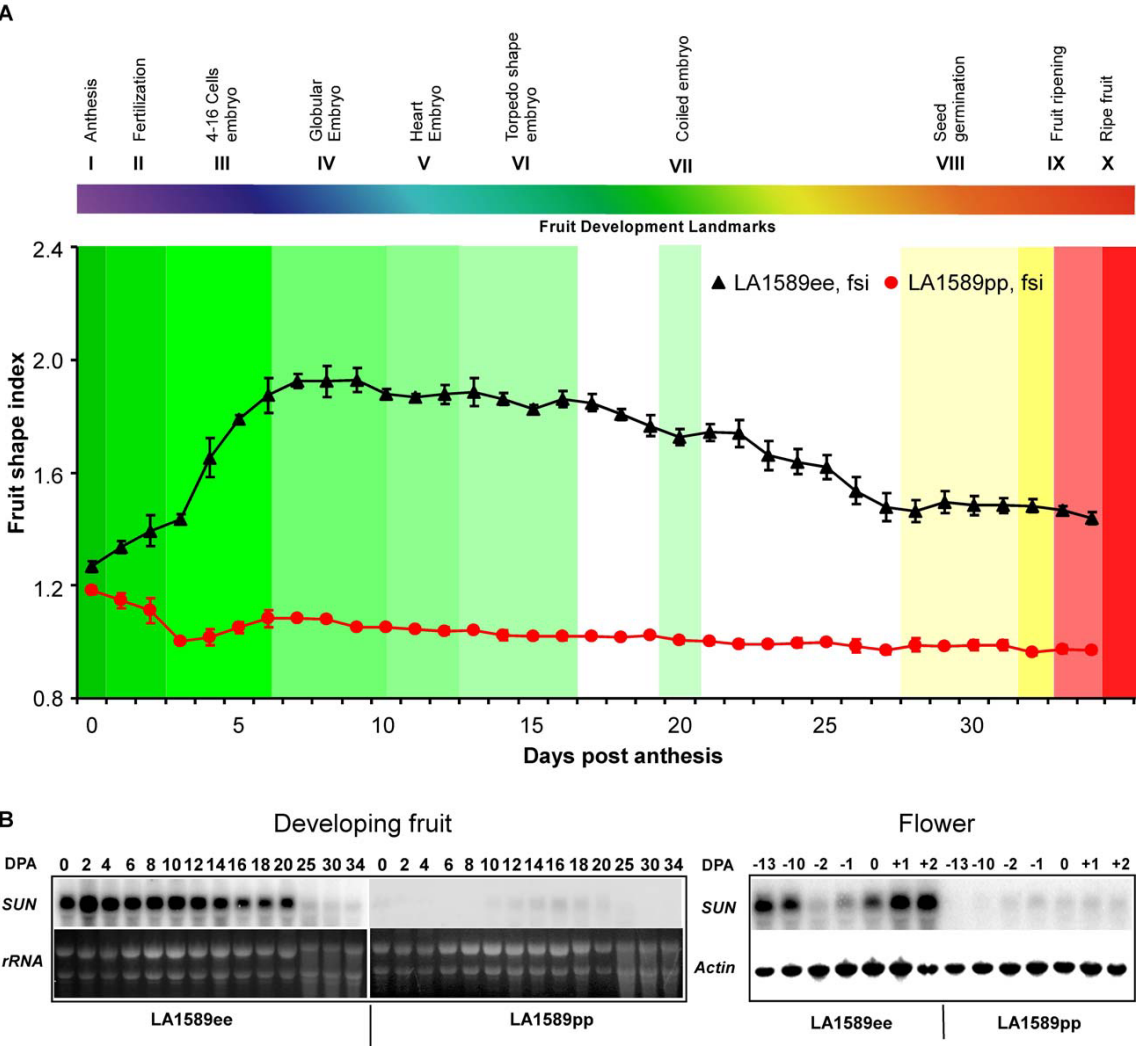
**Fruit developmental landmarks superimposed with shape changes controlled by *SUN***. (A) Fruit landmark stages are color coded and indicated above the graph. Fruit shape index (length/width ratio, Y-axis) is shown as a function of dpa (X-axis). The kinetics of fruit shape change is overlaid on the fruit developmental landmarks. The largest difference in fruit shape indices is achieved at fruit landmark 3 and 4, coinciding with the landmarks 4–16 cell and globular stage of the embryo. Data shown are mean (± se) from three inflorescences per plant and from five plants per genotype. (B) *SUN *expression in the developing fruit and flowers of LA1589 NILs as determined by Northern blot analysis. Tissues were harvested in days post anthesis (DPA) as indicated above the lanes. LA1589ee carries oval shaped fruit and the *sun *allele of Sun1642. LA1589pp carries round fruit and the *sun *allele of LA1589 which is wildtype.

We used the floral and fruit developmental landmarks described above to determine when *SUN *affects tomato fruit shape. *SUN *controls fruit elongation and its high expression results in oval shaped fruit [32]. We analyzed the changes in fruit shape from anthesis onward in NILs in LA1589 background differing at the *sun *locus because at anthesis the ovary shape is only marginally different (Fig. 11). The LA1589pp has round fruit and carries the wild type allele, while LA1589ee carries the Sun1642 allele of *sun *resulting in an elongated fruit (Fig. 11A). The difference in fruit shape between the two NILs became apparent immediately following fertilization and was most pronounced between 6 and 10 dpa coinciding with the globular embryo stage of fruit landmark 4. At the end of the sixth fruit landmark, representing the seed torpedo stage, the fruit shape index of the LA1589ee NIL started to decrease. After the landmark of seed germination corresponding to mature green stage, the fruit shape index remained constant. LA1589pp fruit showed a decrease in fruit shape index from > 1 at anthesis to < 1 at 5 dpa (Fig. 11A). We examined *SUN *expression in the developing fruits of the LA1589 NILs starting from anthesis-stage ovaries until ripe fruit. In LA1589ee, *SUN *was expressed at a high level until fruit landmark 7 coinciding with coiled embryo and seed maturation stage at 20 dpa (Fig. 11B). A detailed investigation of its expression immediately before and after anthesis showed that *SUN *transcript levels increased from 2 days prior to anthesis to 2 dpa and thus showed a similar kinetics to that of the changes in fruit shape (Fig. 11B).

### Gene expression profiles associated with *SUN*

To further investigate the effect of *SUN *on tomato fruit shape and to identify genes that may interact with *SUN *in regulating morphology, we compared transcriptional profiles of three floral and fruit developmental stages in the NILs in LA1589 background that differ at *sun*. The stages selected represented the three important events in flower and early fruit development when *SUN *exhibited the greatest differential gene expression (Fig. 11B), namely 10 days pre-anthesis, anthesis and 5 days post-anthesis fruit. In total, we found 34 differentially expressed genes between the NIL pairs (*p *< 0.05 and fold change FC > 1.4) (see Additional file 4, Table 4). One of the genes, *DEFL2 *encoding a member of plant defensins, was differentially expressed at all three time points. Another gene encoding maternal effect embryo arrest 59 (MEE59, TC125885) was upregulated in oval shaped fruit at two time points. Twenty four genes were differentially expressed only in anthesis-stage flowers and eight genes were differentially expressed only in 5 dpa fruit. The differences in the transcript levels of the 34 genes were less than two-fold with the exception of *DEFL2*. The latter gene is located very close to *SUN *on chromosome 7. Therefore, decreased *DEFL2 *expression in the NIL carrying elongated fruit was likely due to the mutation at the locus and not a consequence of increased expression of *SUN *(see sequence annotation EF094940). The remaining differentially expressed genes did not fall into known developmental pathways. Note that *SUN *and *DEFL1*, which are differentially expressed in these NILs [32] were not on the array.

**Table 4:** Differentially expressed genes in LA1589 *sun *NILs

	Gene ID*	**Fold Changes**	**Gene annotation**	**Category**
Flower bud				
	TC119205	-2.0	Defensin, DEFL2	Defense response
Flower				
	TC119205	-3.4	Defensin, DEFL2	Defense response
	TC123918	-1.6	Pyridoxal 5'-phosphate-dependant histidine decarboxylase	Metabolism
	TC120795	-1.5	Harpin-induced protein 1 (Hin1) (AT5G11890).	Unknown
	TC130586	-1.5	Putative GPI protein (At5g53870)	Energy pathways
	TC118655	-1.5	Unknown	Unknown
	TC129091	-1.5	Weakly similar to potato resistance gene cluster AF265664.	Defense response
	TC119275	-1.5	Auxin-responsive family protein (AT3G25290)	Developmental processes
	TC128245	-1.5	Hypothetical protein	Unknown
	TC131486	-1.5	Hypothetical protein	Unknown
	TC121636	-1.5	Unknown	Unknown
	TC130702	-1.4	Plant thionin family protein (AT1G12663)	Unknown
	TC122761	-1.4	Unknown	Unknown
	TC116706	-1.4	Unknown	Unknown
	TC123023	-1.4	Plastocyanin-like domain-containing protein (AT5G53870)	Energy pathways
	TC126072	1.4	DNAJ-LIKE 20 (At4g13830)	Metabolism
	TC120357	1.4	Universal stress protein (USP) family protein (At3g62550)	Stress response
	TC127119	1.4	Thiamine biosynthesis family protein/thiC family protein (AT2G29630)	Biosynthetic process
	TC124373	1.4	Unknown protein (AT4G32480)	Unknown
	TC131247	1.4	alternative oxidase 2 (AT5G64210)	Energy pathways
	TC116590	1.4	60S ribosomal protein L6 (RPL6A) (AT1G18540)	Biosynthetic process
	TC125885	1.5	MEE59 (maternal effect embryo arrest 59) (AT4g37300)	Developmental processes
	TC127729	1.5	ALPHA-CRYSTALLIN DOMAIN 31.2 (At1g06460 mRNA)	Stress response
	TC116513	1.5	Single-stranded DNA binding protein precursor (AT2G37220)	Stress response
	TC123370	1.6	HEPTAHELICAL TRANSMEMBRANE PROTEIN1 (AT5g20270)	Developmental processes
	TC124422	1.7	Phi-1. Arabidopsis thaliana phosphate-responsive protein (EXO)	Developmental processes
	TC116452	1.8	Pectin methylesterase inhibitor isoform (AT5G62360)	Metabolism
Fruit				
	TC119205	-2.9	Defensin, DEFL2	Defense response
	TC130680	-1.5	unknown	Unknown
	TC116444	-1.4	Auxin/aluminum-responsive protein (AT4G27450)	Unknown
	TC122863	-1.4	Sulfate transporter (AT3G51895)	Transport
	TC122115	1.4	proteinase inhibitor isoform	Stress response
	TC126601	1.4	Gty37 protein; putative cell wall protein (AT2G20870)	Unknown
	TC124142	1.4	2OG-Fe(II) oxygenase family (AT2G36690)	Biosynthetic process
	TC123957	1.4	THI1 protein (AT5G54770)	Biosynthetic process
	TC123969	1.4	Late embryogenesis abundant protein	Developmental processes
	TC125885	1.6	MEE59 (maternal effect embryo arrest 59) (AT4g37300)	Developmental processes

*SUN and *DEFL1 *were not on the Nimblegen Array; genes with RMA expression value smaller than 5 were considered to bee too low expressed and removed from the analysis.

*SUN *has been hypothesized to affect fruit shape by altering hormone levels such as auxin [32]. However, several auxin biosynthesis genes, including *ALDEHYDE OXIDASE 1 *(*AAO1*) and most genes encoding tryptophan biosynthesis enzymes that were present on the array, were not changed in the NILs. Gibberellins (GA) also play important roles in cell division and elongation [75,76]. Similarly, none of the GA biosynthesis genes on the array were differentially expressed. We also performed Northern blots on GA biosynthesis genes that were not on the array and found that none were differentially expressed in the NILs either (data not shown). This implied that *SUN *is not directly involved in regulating auxin and GA levels.

## Discussion

The formation of the flower and fruit can be described by a series of landmarks that coincide with key development events. Floral landmarks described by Buzgo et al. (2004) and fruit landmarks proposed herein provide the framework for comparative analyses of floral and fruit development among angiosperm species. Moreover, understanding the common mechanisms of reproductive development also provides the basis from which to dissect the differences observed among species and the evolution of fruit form [77].

For tomato, *S. pimpinellifolium *accession LA1589 is an excellent model for flower and fruit development because of its predictable growth pattern, large numbers of flowers per inflorescence and inflorescences per plant. Previous studies in cherry tomato (*S. lycopersicum *var. *cerasiforme*) described flower development in 20 stages from sepal initiation to anthesis and established the correlation between major cellular events in reproductive organs with perianth markers [78]. The main floral developmental events we described for LA1589 are in agreement with those observations in cherry tomato, although we started floral development with inflorescence formation and floral initiation rather than sepal initiation. Inflorescence formation and floral initiation is a major event in floral development, and the critical transformation from vegetative meristem to floral meristem is tightly regulated by floral meristem identity genes, such as *LEAFY *and *APETALA1 *[79,80]. Therefore, floral landmark 1 will be of great interest in dissecting functions and expression patterns of floral meristem identity genes in tomato as well as genes that play a role in fruit size and shape. Previous fruit development of cultivated tomato has been divided into phases based on cell division activities [19]. We observed a very short duration of cell division in the pericarp of LA1589 fruit (less than 5 dpa), in contrast to ~7 to 10 dpa in cultivated tomato [19]. Embryogenesis and seed formation in many flowering plants occur concomitantly with fruit development, therefore we described the ontogeny of the fruit following key events in embryogenesis and seed formation. Thus, herein we provide a complete set of consensus landmarks for flower and fruit stages starting from floral initiation until fruit ripening. These landmarks highlight major events in reproductive development and serve as a guide in floral and fruit developmental research. The use of common terminology will make data and information from different species queryable, while also facilitates comparative analysis across species.

Recently, a genome-wide analysis of the transcriptional changes induced by pollination and GA application of ovaries was performed [81]. A comparison between ours and the previously published study showed that some phytohormone related genes were shared in the two studies. Four auxin-related genes, encoding GH3.3 (TC118161), auxin responsive family protein (TC130798), amino acid permease (TC122973) and auxin efflux carrier family protein (TC120936), shared the same expression patterns between the two experiments. However, none of the GA-related genes were shared in the two studies. Abscisic acid (ABA) and ethylene may also play roles in fruit set and fruit growth post pollination as genes involved in biosynthesis and signaling of these phytohormones were differentially expressed after pollination [81]. Similar to the Vriezen et al study (2008), several ACC synthase genes were differentially expressed and all the ethylene biosynthesis genes were less abundant in 5 dpa fruits, suggesting reduced levels of this hormone after pollination. The expression of ABA biosynthesis genes, such as neoxantin synthase (*NSY*) and 9-cis-epoxycarotenoid dioxygenase (*LeNCED*), is reduced in fruits post pollination [81]. Similarly, in our study zeaxanthin epoxidase (*ZEP/ABA1*) was less abundant in 5 dpa fruit compared to flower. In both studies, an *ABA 8'-hydroxylase *gene (cytochrome P450 family member) involved in ABA catabolism [74], was more abundant in fruits post pollination. This suggests that ABA, like ethylene, is in low demand during fruit set and early growth. Recently, Galpaz et al (2008) determined that tomato *high-pigment 3 *(*hp3*) mutant with a mutation in the *ZEP *gene produces a higher level of fruit lycopene linked to increased plastid number as a result of ABA deficiency [82]. Because the *hp3 *mutant makes smaller fruit [82], certain amounts of ABA may be required for fruit growth after anthesis.

Transcriptional profiles of other classes of genes were also similar between the previously published study [81] and ours. More than half (13 of 22) of cell cycle-related genes and half (13) of the cell wall-related genes were shared between the two studies (see Additional file 5) [81]. Two cyclin genes TC120949 and TC128804, showing highest similarities to Arabidopsis *CYCLIN D3;1 *(*CYCD3;1*) and *CYCLIN B1;4 *(CYCB1;4), were induced by pollination, but not by GA treatment based on previous observations [81]. However, their higher expression before and after anthesis in our experiments suggests that the two genes are not only inducible by pollination but also involved in pre-anthesis activation of cell division possibly in response to other hormone cues such as cytokinin. In Arabidopsis, *CYCD3;1 *responds to cytokinin to activate cell division at the G1-S cell cycle phase [83].

After establishing the morphological landmarks for flower and fruit development in tomato, we superimposed the effect of *SUN *on fruit formation. *SUN *controls fruit shape after anthesis [32]. From the landmark fertilization to the landmark globular embryo stage, the fruit shape index dramatically increased in the accession that expresses *SUN *to a high level (Fig. 11). The coincidence between the dynamics of fruit shape index mediated by *SUN *and fruit growth suggests that *SUN *mainly acts in fast growing tissues, which is further supported by high expression of *SUN *in the oval shaped fruits during early fruit growth. Although we hypothesized that SUN may indirectly affect hormone or secondary metabolite levels and as such altering organ shape [32], the identified differentially expressed genes did not support that notion. Moreover, the very low number of differentially expressed genes was surprising considering that the expression of *SUN *was quite high in the lines carrying oval-shaped fruit at the time points sampled.

## Conclusion

Following the universal landmarks proposed by Buzgo et al (2004), we outlined flower and fruit developmental landmarks in tomato. Transcriptional profiles of flower and developing fruit at three main stages have been integrated with their corresponding landmarks, which will be useful for identifying important regulatory components responsible for key developmental processes. We identified genes encoding patterning, phytohormone and cell cycle-related proteins modulated during flower and early fruit development, which will provide basis for further studies on tomato fruit growth. The usefulness of the landmarks was demonstrated by examining the fruit shape changes mediated by *SUN*.

## Methods

### Plant materials

Seeds of *S. pimpinellifolium *accession LA1589 were obtained from the C.M. Rick Tomato Genetics Resource Center, Davis, California, USA. Nearly Isogenic Lines (NILs) that differ at *sun *locus were resulted from the high-resolution recombinant screens conducted to fine map the locus [84]. After multiple backcrosses and molecular marker analysis, we estimated that the introgression of the Sun1642 allele in the LA1589 background is less then 100 kb with very few, if any, other regions of the genome harboring the Sun1642 allele. Plants were grown under standard conditions with supplemental lighting in the greenhouse.

### Timing of flower opening on individual inflorescences

Eighty three inflorescences from four independent experiments were tagged before flower opening. Anthesis was recorded each day at the same time, and two flowers that opened on the same day were recorded as 0 days between flowerings.

### Seed viability determination

Seeds were extracted from the fruit harvested on tagged inflorescences that were hand pollinated to ensure uniform fruit set. The dates of pollination were recorded and the fruits were harvested based on days after anthesis. Seeds were extracted and incubated for 20 min in 25% HCl to remove the gelatinous layer surrounding the seed, rinsed with distilled water and germinated for one week in the dark at 30°C on moist Whatman paper.

### Ethylene sensitivity of developing fruit

Tagged flowers were hand pollinated and the dates were recorded. Fifteen to 20 fruit from mature green to breaker (26–33 dpa) were treated for 16 hours in a sealed chamber with 10 μl/L ethylene and the color changes were monitored two days later. Color for each fruit was recorded into different categories (green, color turning, orange, yellow and red) before and after ethylene treatment, and ethylene sensitivity was expressed by fruits with changed colors in total fruits assayed.

### Timing of fertilization

Flowers were emasculated one day prior to anthesis and hand-pollinated the next day. Pistils were collected at 6, 8, 10, 12 and 24 hours after pollination. Dissected pistils were fixed in 3:1 95% ethanol:glacial acetic acid overnight at room temperature. Samples were subsequently softened for 24 hours in 10 N NaOH, rinsed five times in ddH_2_O and stained using 0.1% aniline blue (aniline blue fluorochrome, Biosupplies Australia) in 0.1 M potassium phosphate buffer pH8.0 for 4 hours in the dark. Samples were mounted in 30% glycerol and viewed on a Leica DM IRB epifluorescence microscope using the UV filter set (Chroma filter A, BP340-380, LP425).

### Fruit shape changes during development

Data were collected from five individual NIL plants per genotype homozygous for *sun*. For ovary and developing fruits from anthesis to 34 dpa, developing fruit were cut in half longitudinally and images were obtained using camera connected to dissection microscope (0–7 dpa) or using scanner (fruit older than 7 dpa). Shape index (length divided by width) were obtained with ImageJ  on images taken. Each time point has at least three ovaries or fruits from each individual plant.

### Scanning electron microscopy of floral development

Flowers were processed in its entirety or partially dissected under the dissecting microscope prior to fixation. Samples were infiltrated and fixed with 3% gluteraldehyde, 2% paraformaldehyde in 0.1 M potassium phosphate buffer pH7.4 for two hours at room temperature and then over night at 4°C. After 3 washes with ddH_2_O samples were post fixed with 1% osmium tetroxide, washed 3 times with ddH_2_O and dehydrated following a graded ethanol series (once for 25%, 50%, 70%, 90%, twice 100%). Critically point dried (Samdri-790, Tousimis Research Corporation) samples were mounted on aluminum stubs, and sputter-coated with platinum (Polaron). When necessary, flower buds were further dissected after platinum coating. Samples were viewed and images recorded with a Hitachi 3500N scanning electron microscope under high vacuum.

### Light microscopy

Flower and fruit samples were infiltrated and fixed in 3% glutaraldehyde, 4% paraformaldehyde, 0.05% Triton X-100 in 0.1 M potassium phosphate buffer at pH 7.2 for two hours at room temperature and then over night at 4°C. After three washes with potassium phosphate buffer, samples were processed for embedding into London Resin White (LRW) (EMS) or paraffin (PolyFin, Polyscience).

For LRW embedding, samples were dehydrated in a graded ethanol series (25%, 50%, 70%, twice 90%), infiltrated with a graded resin and 90% ethanol series (1:3, 1:1, 3:1, twice 100% resin), embedded in airtight gelatine capsules (EMS) and polymerized overnight at 60°C. Five μm thick sections were collected on glass slides and stained with 0.1% sodium bicarbonate, 0.5% toluidine blue, in 25% EtOH before light microscopy observation.

For paraffin embedding, samples were dehydrated in a graded ethanol series (50%, 80%, 90% twice 100%), and subsequently infiltrated, first in a graded ethanol/tertiary butyl alcohol (TBA) series at room temperature (2:1, 1:1, 1:2, twice 100% TBA), and then in a graded TBA/paraffin series (1:3, 1:1, 3:1, twice 100% paraffin) at 56°C and embedded in paraffin. 6–10 μm sections were collected on silane treated glass slides (Polyscience). Deparaffinized sections were stained 10 minutes with 10 mg/ml safranin O in 50% ethanol, and 10 seconds with 5 mg/ml astra blue containing 20 mg/ml tartaric acid following Jensen procedure [85]. Sections were observed on the Leica DM IRB light microscope (Leica Microsystems, Wetzlar Germany) and images were captured using the MagnaFire model S99802 digital camera (Optronics, Goleta, CA).

For fluorescent microscopy, sections were deparaffinized, blocked with 10 mM potassium phosphate buffer (pH7.4), 150 mM NaCl (PBS) containing 10 mM NaAzide, 0.2%BSA, 1% normal goat serum for 30 minutes. Tubulin was detected using a 1/500 dilution of the mouse anti-tubulin monoclonal IgG1 (Molecular Probes) as primary antibody, and AlexaFluor488 sheep anti-mouse secondary antibody (Invitrogen, USA). Antibody incubations were performed in incubation buffer (PBS containing 10 mM NaAzide, 0.2%BSA) at room temperature for 4 hours for the primary antibody, and 2 hours for the secondary antibody. After each incubation, the sections were washed five times with PBS. Cell nuclei were counterstained for 8 minutes with 0.25 mM SytoxOrange (Invitrogen, USA). Sections were then mounted with GelMount (Biomedia) and observed on a Leica TCS-NT confocal microscope.

Additional developing embryos were visualized using differential interference contrast microscopy. Samples were fixed in 10:3:1 ethanol, glacial acetic acid, chloroform mixture. Tissue was rinsed in 90% ethanol twice, and then cleared in modified Hoyer's solution consisting of 60 ml of distilled water, 7.5 g arabic gum, 100 g chloral hydrate, 5 ml of glycerin. Samples were mounted in 70% glycerol, smashed using the cover slip and viewed with a Nomarski objective or phase contrast using the Leica DM IRB light microscope.

### Pericarp cell number and size measurements

Fruits were harvested at 0, 2, 5, and 10 dpa. Prior to fixation, fruit of 5 and 10 dpa were cut longitudinally and perpendicular to the septum, while fruit of 0 and 2 dpa were fixed as a whole. The fixed tissues were embedded into London Resin White as described above. Sections were collected from 6 and 20 samples per time point. Pericarp consists of epicarp (the single outermost cell layer), endocarp (the single innermost cell layer) and mesocarp comprising of cells in-between epicarp and endocarp. Cell lengths of epicarp and endocarp were determined by averaged lengths of 5–10 cells along. The length of the mesocarp was measured in the middle region of the mesocarp sampling 5–10 cells. Cell volume was calculated based on formula V = L*W*((L+W)/2), where V represents cell volume, L = cell length, W = cell width.

### Microarray analysis

The tomato microarray was custom-designed oligoarray manufactured by Nimblegen (Nimblegen Inc. USA) based on TIGR tomato Tentative Contigs sequences (release 9, ). It consists of 15270 TCs corresponding to 7600 different clusters (transcripts) and each TC was represented by 12 pairs of perfect and mismatch probes of 24-mers.

Total RNA for microarray analysis were extracted from 10-day preanthesis flower bud and anthesis flower and fruits at 5 dpa using Trizol reagent (Invitrogen Inc. USA). Before RNA extraction, tissues harvested at 7-day interval from five plants were pooled for each genotype. Three biological replicates were conducted with three sets of LA1589 *sun *NILs growing during different time periods resulting in 3 time points × 2 genotypes × 3 replicates = 18 array hybridizations. Microarray hybridizations, image scanning and data extracting were performed by Nimblegen Inc. Background correction and data normalization were performed by Robust Microarray Analysis (RMA, [86]) in Bioconductor. Differentially expressed genes (DEs) among the three stages were selected by multiple testing package *multtest *[40] of R  using the RMA expression values. To update the gene description and annotation, sequences of the differentially expressed genes were BLASTed against Arabidopsis protein database (version 7 released on July 24, 2007 by TAIR, .) using blastx. Description of proteins encoded by some differentially expressed genes with low homology (*p *< 1e-10) to Arabidopsis proteins was assigned with the annotation of the newest TC (release version 11 by TIGR) or those with best hit in NCBI database . The data discussed in this publication have been deposited in NCBI's Gene Expression Omnibus [87] and are accessible through GEO Series accession number GSE15453 .

### Northern blot

RNA was isolated from the whole fruit or flower using Trizol reagent (Invitrogen Inc. USA) (for ovary and fruits of 20 dpa or younger), or the hot borate method (for fruits of 25, 30, and 34 dpa old) [88]. For Northern blot, 10 *μ*g of the total RNA of each sample was separated in 1.2% Agarose gel in 1XMOPS buffer with formaldehyde, transferred onto Hybond N membrane (Amersham Biosciences) and hybridized at 42°C in formamide-containing hybridization buffer with radiolabeled gene-specific probes sequentially after previous probes were stripped.

## Authors' contributions

HX and NW conducted the experiments on ethylene and seed germination, and fruit shape mediated by *SUN*. HX conducted the Northern blots and together with JH and DL the transcript profiling analysis. CR and TM conducted the floral landmark study. NW and TM conducted the fruit landmark study. EvdK supervised the project and conducted the pollination experiment. HX, TM, NW and EvdK wrote the paper with editorial comments from the other authors.

## Supplementary Material

Additional file 1 **Differentially expressed genes at 10 day pre-anthesis flower, anthesis flower and 5 dpa fruit**. The spreadsheet contains all the transcripts that were differentially expressed between any of the three stages: 10 days pre-anthesis flower buds (pre), anthesis flowers (ant) and 5 dpa fruit (post). Click here for file

Additional file 2 **Diffentially expressed genes involved in developmental processes**. The spreadsheet contains the differentially expressed transcripts that encode proteins involved in developmental processes. Click here for file

Additional file 3 **Differentially expressed genes related to phytohormones**. The spreadsheet contains the differentially expressed transcripts that encode proteins involved in biosynthesis, signal perception or transport of phytohormones. Click here for file

Additional file 4 **Plot of RMA intensities between LA1589 NILs that differ at *sun***. Background collection and normalization of hybridization signals were performed on the 18 arrays using the Robust Multichip Average algorithm (RMA). (A) Plot of RMA values for flower buds at 10 days preanthesis (Bud). (B) Plot of RMA values for anthesis-stage flower (Flower). (C) Plot of RMA values for 5 dpa fruit (Fruit). Data shown in the graphs are averaged log_2_(RMA intensities) of the three biological replicates. The number in each plot is the Pearson's coefficient (*R*) between the two genotypes, LA1589ee, homozygous LA1589 NILs with *SUN*, and LA1589pp, homozygous for the wild-type allele. Click here for file

Additional file 5 **Differentially expressed genes involved in the cell cycle and cell wall modification**. The spreadsheet contains the differentially expressed transcripts that encode proteins involved in regulation of cell cycle and cell wall modification. Click here for file
