# Supplementary material for: Integration of tomato reproductive developmental landmarks and expression profiles, and the effect of SUN on fruit shape
Source: BMC Plant Biol. 2009 May 7;9:49. doi: 10.1186/1471-2229-9-49 (PMC2685393; doi:10.1186/1471-2229-9-49)
Supplement: Additional file 4 — Plot of RMA intensities between LA1589 NILs that differ at sun. Background collection and normalization of hybridization signals were performed on the 18 arrays using the Robust Multichip Average algorithm (RMA). (A) Plot of RMA values for flower buds at 10 days preanthesis (Bud). (B) Plot of RMA values for anthesis-stage flower (Flower). (C) Plot of RMA values for 5 dpa fruit (Fruit). Data shown in the graphs are averaged log2(RMA intensities) of the three biological replicates. The number in each plot is the Pearson's coefficient (R) between the two genotypes, LA1589ee, homozygous LA1589 NILs with SUN, and LA1589pp, homozygous for the wild-type allele. [file 1471-2229-9-49-S4.doc]

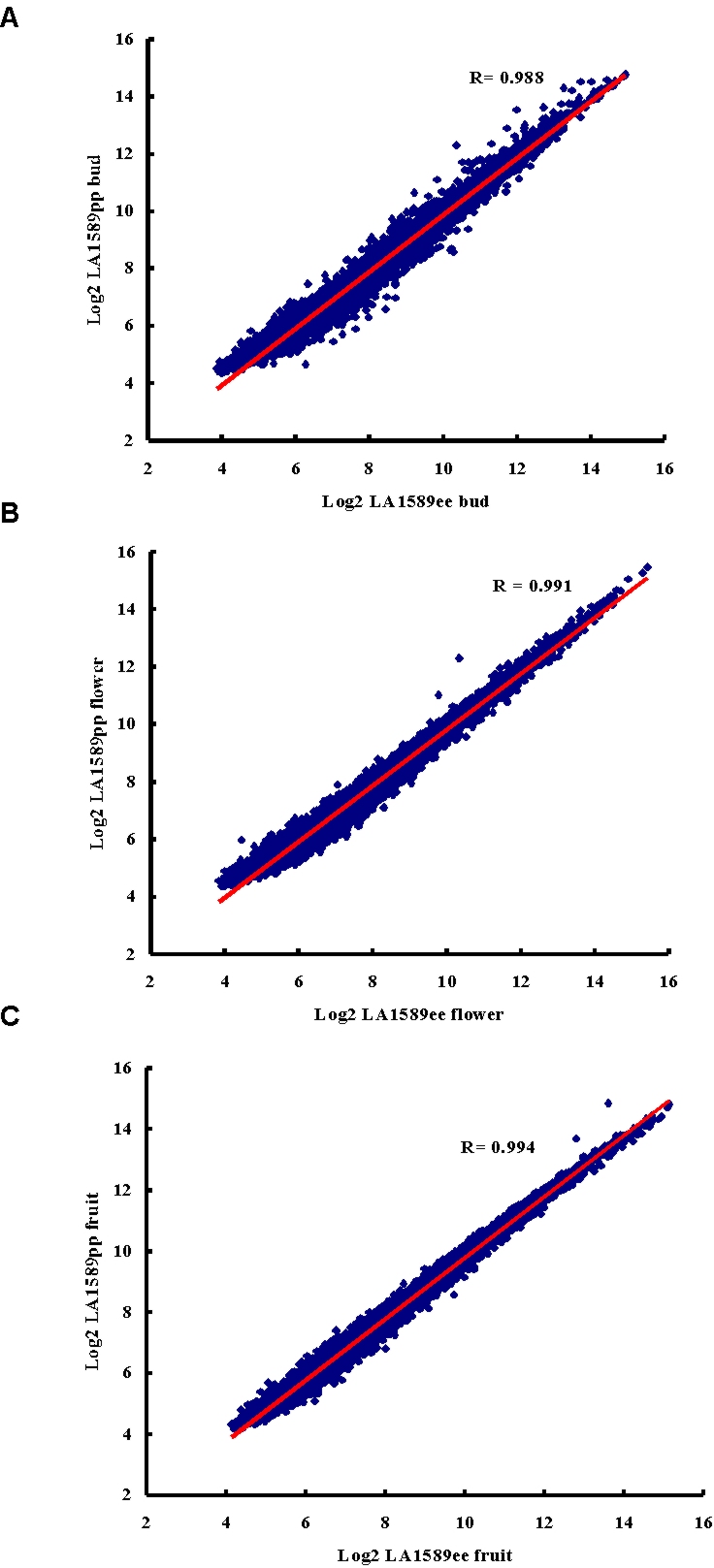


Additional file 4 Plot of RMA intensities between LA1589 NILs that differ at *sun*

Background collection and normalization of hybridization signals were performed on the 18 arrays using the Robust Multichip Average algorithm (RMA). (A) Plot of RMA values for flower buds at 10 days preanthesis (Bud). (B) Plot of RMA values for anthesis-stage flower (Flower). (C) Plot of RMA values for 5 dpa fruit (Fruit). Data shown in the graphs are averaged log_2_(RMA intensities) of the three biological replicates. The number in each plot is the Pearson’s coefficient (*R*) between the two genotypes, LA1589ee, homozygous LA1589 NILs with *SUN,* and LA1589pp, homozygous for the wild-type allele
